# Inhibition of Melanogenesis by Essential Oils from the Citrus Cultivars Peels

**DOI:** 10.3390/ijms24044207

**Published:** 2023-02-20

**Authors:** Jiyoon Yang, Su-Yeon Lee, Soo-Kyeong Jang, Ki-Joong Kim, Mi-Jin Park

**Affiliations:** 1 Forest Industrial Materials Division, Forest Products and Industry Department, National Institute of Forest Science, Seoul 02455, Republic of Korea; 2 Division of Life Sciences, Korea University, Seoul 02841, Republic of Korea

**Keywords:** citrus, essential oil, natural products, anti-melanogenesis, β-elemene, farnesene, limonene

## Abstract

Citrus is one of the most popular and widely grown fruit crops in the world. However, the bioactivity of only certain species of citrus cultivars is studied. In this study, the effects of essential oils from 21 citrus cultivars on melanogenesis were investigated in an effort to identify active anti-melanogenesis constituents. The essential oils from the peels of 21 citrus cultivars obtained by hydro-distillation were analyzed using gas chromatography–mass spectrometry. Mouse melanoma B16BL6 cells were used in all assays conducted in this study. The tyrosinase activity and melanin content were determined using the lysate of α-Melanocyte-stimulated B16BL6 cells. In addition, the melanogenic gene expression was determined by quantitative reverse transcription-polymerase chain reaction. Overall, the essential oils of (*Citrus unshiu* X *Citrus sinensis*) X *Citrus reticulata*, *Citrus reticulata*, and ((*Citrus unshiu* X *Citrus sinensis*) X *Citrus reticulata*) X *Citrus reticulata* provided the best bioactivity and comprised five distinct constituents compared to other essential oils such as limonene, farnesene, β-elemene, terpinen-4-ol, and sabinene. The anti-melanogenesis activities of the five individual compounds were evaluated. Among the five essential oils, β-elemene, farnesene, and limonene showed dominating properties. The experimental results indicated that (*Citrus unshiu* X *Citrus sinensis*) X *Citrus reticulata*, *Citrus reticulata*, and ((*Citrus unshiu* X *Citrus sinensis*) X *Citrus reticulata*) X *Citrus reticulara* are potential candidates with anti-melanogenesis activity for use as cosmetics and pharmaceutical agents against skin hyperpigmentation.

## 1. Introduction

The genus *Citrus* comprises some of the most significant fruit trees that are cultivated worldwide [1]. *Citrus* is in the subfamily Aurantioideae belonging to the family Rutaceae, which is widely distributed in tropical regions and comprises about 160 genera and 1800 species [2]. In Korea, the Jeju Citrus Federation has reported that domestic citrus production has increased steadily since 2017 and reached about 655,000 tons in 2020, among which the satsuma accounts for 572,000 tons and late-maturing species account for 82,000 tons. Citrus fruits contain various nutrients such as vitamins, folate, and potassium, as well as secondary metabolites such as essential oils, flavonoids, alkaloids, carotenoids, and phenolic acids [3]. A lot of studies have focused on these phytochemicals of citrus cultivars and their various beneficial effects, such as their antioxidant, anti-inflammatory, anti-cancer, and anti-microbial activities [4].

Melanocytes are commonly found in human skin, hair, and eyes and are synthesized in organelles called melanosomes [5]. α-Melanocyte-stimulating hormone (α-MSH), which is secreted from the pituitary gland, is a pigmentary hormone that promotes melanogenesis [6]. α-MSH binds to melanocortin receptor 1 (*MC1R*) on the melanocyte surface, which leads to elevated intracellular cyclic adenosine monophosphate (cAMP) and microphthalmia-associated transcription factor (*MITF*) expression [7]. MITF regulates the transcription of melanogenic genes including tyrosinase, tyrosinase-related protein 1 (*TRP-1*), and tyrosinase-related protein 2 (*TRP-2*) by binding to promoter regions [8]. Tyrosinase is a rate-limiting enzyme that plays an important role in melanin synthesis in melanocytes by catalyzing the conversion of L-tyrosine to L-3,4-dihydroxyphenylalanine (DOPA) and thereafter to DOPA chrome, which is subsequently polymerized to form melanin [9]. Melanin is a protective pigment in the skin against UV radiation, but excessive deposition can cause many skin disorders [10]. Therefore, tyrosinase inhibitors are being researched to inhibit melanin pigmentation.

Tyrosinase inhibitors such as hydroquinone, kojic acid, and arbutin are used as whitening cosmetics or pharmaceuticals but have side effects [11]. Hydroquinone is the most common treatment for hyperpigmentation but is potentially mutagenic to cells and is associated with a number of side effects such as irritation, contact dermatitis, and transient erythema [12]. Kojic acid may be carcinogenic and may cause contact dermatitis. Arbutin is chemically unstable and is potentially genotoxic, and ascorbic acid is easily degraded by exposure to oxygen, high temperature, and high pressure [13,14]. Natural products are known to have low side effects [15]. Thus, this study aimed to find compounds with superior anti-melanogenesis activity among natural products with low side effects.

Recently, interest has increased in studying whether medicinal plants and their bioactive components can suppress hyperpigmentation. The extracts from *Azadiracta indica, Curcuma longa, Embica officinalis, Glycyrrhiza glabra,* and *Panax ginseng* have been known to be effective against skin hyperpigmentation [16]. In addition, components isolated from plant extracts such as epicatechin-3-gallate, linolenic acid, and Arctigenin, epiceanothic acid were involved in the modulation of melanogenesis [17]. Essential oils are a key constituent of medicinal plants that have been used since ancient times and are enormously valuable for various physiological applications [18]. Various essential oils are widely used for treating physiological dysfunctions of the skin, including hyperpigmentation, acne, skin aging, dry skin, eczema, and dermatitis [19]. Essential oils from *Alpinia zerumbet*, *Eucalyptus camaldulensis*, and *Calocedrus formosana* are used to alleviate excessive pigmentation conditions such as skin spots [20,21,22]. Citrus fruits contain compounds with anti-melanogenesis activities that are potentially suitable for use in whitening cosmetics and hyperpigmentation treatments [23]. The essential oils in citrus fruits are known for their cosmetic efficacy, but their anti-melanogenesis activity has not been intensively studied. In addition, the studies on the anti-melanogenesis activity of citrus essential oils have been limited to some species of cultivars. In this study, essential oils were extracted from 21 citrus cultivars and their anti-melanogenesis activity was evaluated. The active compounds of essential oils that showed the best anti-melanogenesis activities were identified for potential application in whitening cosmetics.

## 2. Results

### 2.1. Chemical Composition

**Figure 1:**
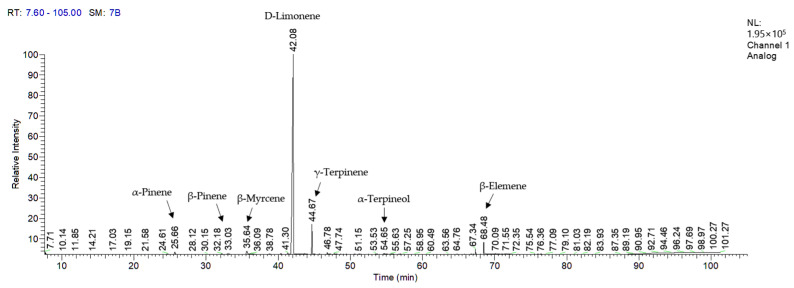
Total ion chromatogram of essential oils from *Citrus reticulata* using GC–MS.

Retention indices based on the Kovats retention index (KI) are robust constants that convey distinct information about the compounds they describe. Therefore, they are utilized to identify volatile compounds in complex GC chromatograms [24]. The total ion chromatogram recorded via the GC–MS of essential oils from 21 citrus cultivars is shown in Figure 1, and the chemical composition of the essential oils is summarized in Table 1 only for components with a content of 1% or more.

The essential oils comprised monoterpene hydrocarbons (83.69–98.95%), oxygenated monoterpenes (0.52–9.10%), sesquiterpene hydrocarbons (0.02–1.46%), oxygenated sesquiterpenes (0–1.17%), and unknown compounds (0.08–1.00%). The essential oils from citrus cultivars contained nine compounds in common; α-pinene, D-limonene, trans-β-ocimene, γ-terpinene, terpinolene, linalool, β-terpineol, terpinene-4-ol, and α-terpineol. D-limonene (50.88–97.19%) was an abundant component in all 21 essential oils. Other major components included γ-terpinene, β-myrcene, α-terpineol, and β-pinene. The essential oils also contained trace elements such as sabinene, β-elemene, and β-farnesene.

The essential oils of 21 citrus cultivars were similar in chemical composition, but they could be classified into four groups by examining five key components (sabinene, β-pinene, β-myrcene, (Z)-citral, β-elemene). Each group contained the original species (mandarin, citron, pummelo, and kumquat). The component differences between these essential oil groups are believed to cause a difference in anti-melanogenesis activity.

### 2.2. Effects of Essential Oils

#### 2.2.1. Cell Viability

**Figure 2:**
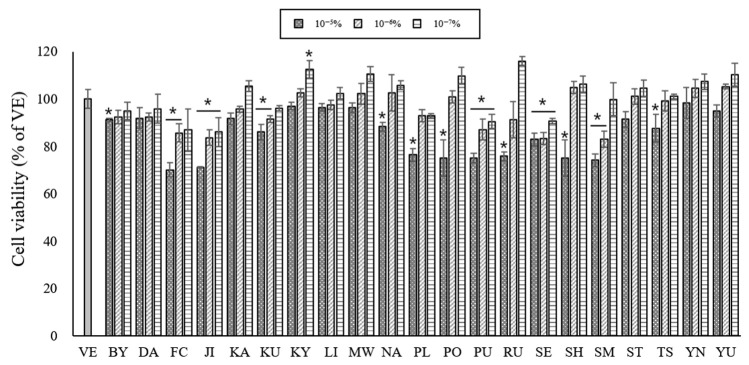
Cell viability assessed by CCK assay. Values are presented as means ± standard deviations. Note: * *p* < 0.05 compared to VE. VE, vehicle group; BY, *C. platymamma*; DA, *C. maxima*; FC, *C. medica*; JI, *C. sunki*; KA, C. X *aurantium*; KU, *C. japonica*; KY, *C. unshiu* X *C. sinensis*; LI, *C. limon*; MW, *C. reticulata*; NA, C. X *aurantium*; PL, *C.* X *latifolia*; PO, *C. reticulata*; PU, *C. maxima*; RU, *C*. *paradisi*; SE, C. X *aurantium*; SH, (C. unshiu X C. sinensis) X C. reticulata; SM, C. reticulata; ST, ((*C. unshiu* X *C. sinensis*) X *C. reticulata*) X *C. reticulata*; TS, (*C. unshiu* X *C. sinensis*) X *C. unshiu*; YN, *C. sinensis*; YU, *C. junos*.

To assess the effects of the 21 essential oils on cell viability, B16BL6 mouse melanoma cells were treated with various concentrations of essential oils (10^−7^–10^−5^%) for 24 h. Figure 2 shows that the cell viability was >80% at concentrations of 10^−7^–10^−6^%, and almost no cytotoxicity was observed at concentrations below 10^−6^%. Thus, to evaluate the anti-melanogenesis effects of the essential oils, B16BL6 melanoma cells were treated with essential oils at concentrations of 10^−6^%.

#### 2.2.2. Tyrosinase Activity

**Figure 3:**
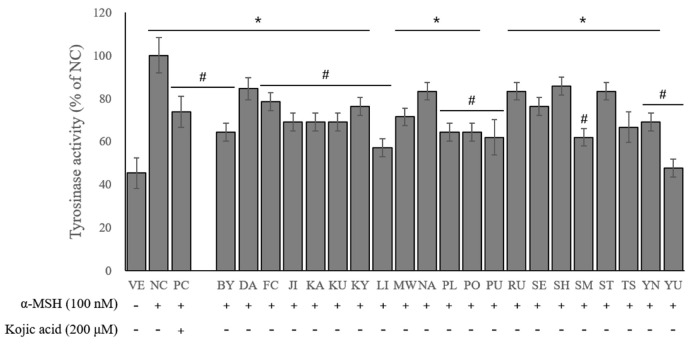
Effects of essential oils on tyrosinase activity. Values are presented as means ± standard deviations. Note: * *p* < 0.05 compared to VE; # *p* < 0.05 compared to NC. VE, vehicle group; NC, 100 nM α-MSH; PC, 200 µM kojic acid; BY, *C. platymamma*; DA, *C. maxima*; FC, *C. medica*; JI, *C. sunki*; KA, *C.* X *aurantium*; KU, *C. japonica*; KY, *C. unshiu* X *C. sinensis*; LI, *C. limon*; MW, *C. reticulata*; NA, *C.* X *aurantium*; PL, *C*. X *latifolia*; PO, *C. reticulata*; PU, *C. maxima*; RU, *C. paradisi*; SE, *C*. X *aurantium*; SH, (*C. unshiu* X *C. sinensis*) X *C. reticulata*; SM, *C. reticulata*; ST, ((*C. unshiu* X *C. sinensis*) X *C. reticulata*) X *C. reticulata*; TS, (*C. unshiu* X *C. sinensis*) X *C. unshiu*; YN, *C. sinensis*; YU, *C. junos*.

The tyrosinase activity was measured using L-DOPA as a colorimetric substrate. Figure 3 shows that the α-MSH-stimulated cells treated with kojic acid and essential oils significantly suppressed the tyrosinase secretion, which demonstrated their anti-melanogenesis activity.

In the negative control (NC) group (i.e., α-MSH stimulation only), the tyrosinase secretion was about 2.2-fold higher than that of the vehicle (VE) group. However, the treatment with kojic acid significantly decreased the tyrosinase secretion compared with the NC group by 26.2%. Similarly, the treatment with essential oils significantly reduced the tyrosinase secretion by 14.3–52.4%. Consequently, the 21 essential oils exhibited considerable anti-melanogenesis activity, among which C. junos (52.4%) and C. limon (42.9%) were the most potent.

#### 2.2.3. Melanin Content

**Figure 4:**
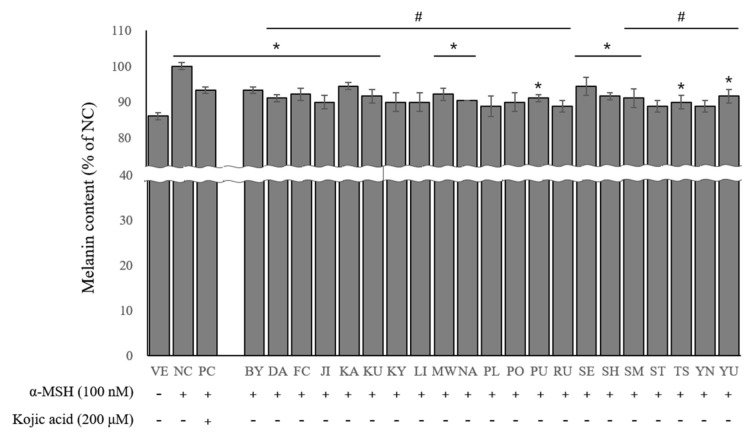
Effects of essential oils on melanin content. Values are presented as means ± standard deviations. Note: * *p* < 0.05 compared to VE; # *p* < 0.05 compared to NC. VE, vehicle group; NC, 100 nM α-MSH; PC, 200 µM kojic acid; BY, *C. platymamma*; DA, *C. maxima*; FC, *C. medica*; JI, *C. sunki*; KA, *C.* X *aurantium*; KU, *C. japonica*; KY, *C. unshiu* X *C. sinensis*; LI, *C. limon*; MW, *C. reticulata*; NA, *C.* X *aurantium*; PL, *C.* X *latifolia*; PO, *C. reticulata*; PU, *C. maxima*; RU, *C. paradisi*; SE, *C.* X *aurantium*; SH, (*C. unshiu* X *C. sinensis*) X *C. reticulata*; SM, *C. reticulata*; ST, ((*C. unshiu* X *C. sinensis*) X *C. reticulata*) X *C. reticulata*; TS, (*C. unshiu* X *C. sinensis*) X *C. unshiu*; YN, *C. sinensis*; YU, *C. junos*.

To determine whether the essential oils of the 21 citrus cultivars inhibited melanogenesis in the B16BL6 cells, the melanin content was measured after 48 h of treatment with kojic acid and essential oils. Figure 4 illustrates the inhibitory effect of the essential oils on melanin production. The melanin content increased following stimulation with α-MSH compared to the VE group. Melanin production was inhibited at similar levels by treatment with essential oils (5.06–11.2%) and kojic acid (6.7%). In particular, *C.* X *latifolia*, *C. paradisi*, ((*C. unshiu* X *C. sinensis*) X *C. reticulata*) X *C. reticulata*, and *C. sinensis* had a greater inhibitory effect than the other essential oils.

#### 2.2.4. Gene Expression

**Figure 5:**
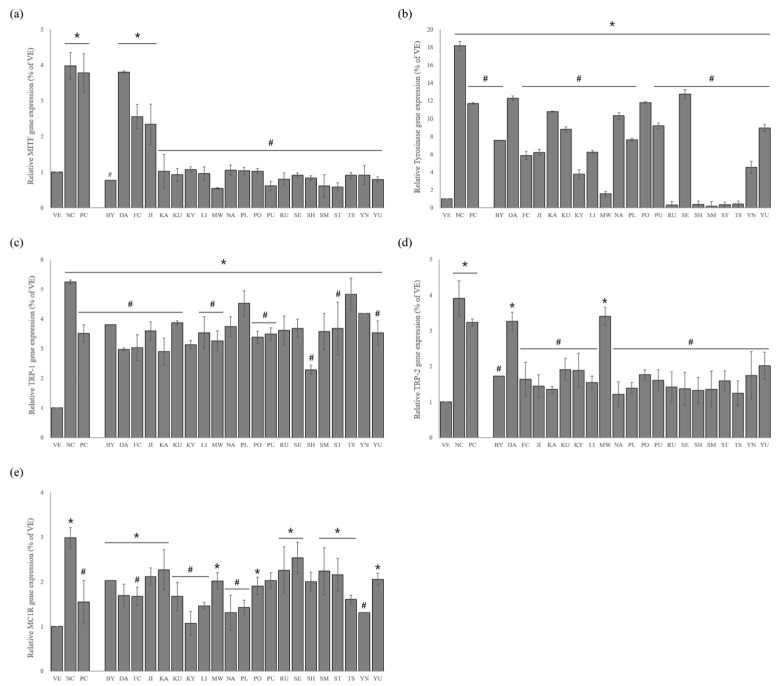
Effects of essential oils on *α*-MSH-induced B16BL6 cells: (**a**) *MITF* expression; (**b**) *Tyrosinase* expression; (**c**) *TRP-1* expression; (**d**) *TRP-2* expression; (**e**) *MC1R* expression. Values are presented as means ± standard deviations. Note: * *p* < 0.05 compared to VE; # *p* < 0.05 compared to NC. VE, vehicle group; NC, 100 nM α-MSH; PC, 200 µM kojic acid; BY, *C. platymamma*; DA, *C. maxima*; FC, *C. medica*; JI, *C. sunki*; KA, *C.* X *aurantium*; KU, *C. japonica*; KY, *C. unshiu* X *C. sinensis*; LI, *C. limon*; MW, *C. reticulata*; NA, *C.* X *aurantium*; PL, *C.* X *latifolia*; PO, *C. reticulata*; PU, *C. maxima*; RU, *C. paradisi*; SE, *C.* X *aurantium*; SH, (*C. unshiu* X *C. sinensis*) X *C. reticulata*; SM, *C. reticulata*; ST, ((*C. unshiu* X *C. sinensis*) X *C. reticulata*) X *C. reticulata*; TS, (*C. unshiu* X *C. sinensis*) X *C. unshiu*; YN, *C. sinensis*; YU, *C. junos*.

A qRT–polymerase chain reaction (PCR) assay was performed to investigate the effects of the essential oils from 21 citrus cultivars in regulating the expression level of melanogenesis genes. As shown in Figure 5, the α-MSH stimulation significantly upregulated the expression of melanogenic genes such as *TRP-1*, *TRP-2*, *Tyrosinase*, *MC1R*, and transcription factor *MITF*. The effects of the essential oils were investigated in terms of the mRNA expression of *MITF*. The NC group showed an approximately 4.0-fold increase in *MITF* expression compared to the VE group (Figure 5a). In contrast, the treatment with kojic acid suppressed the *MITF* expression compared to the NC group. The essential oils also inhibited the *MITF* expression, but were more active than the kojic acid (5.0% reduction). Furthermore, superior inhibitory activity was observed for *C. reticulata* (MW, 86.4% reduction), ((*C. unshiu* X *C. sinensis*) X *C. reticulata*) X *C. reticulata* (85.3%), *C. reticulata* (SM, 84.7%), and *C. maxima* (PU, 84.5%).

The NC group showed a remarkable increase in Tyrosinase expression compared with the VE group (Figure 5b), whereas the *Tyrosinase* expression level was significantly reduced after treatment with kojic acid and essential oils. The treatment with kojic acid decreased the *Tyrosinase* expression by about 35.6% compared to the NC group. The treatment with the 21 essential oils also significantly reduced the *Tyrosinase* expression compared to the NC group. In particular, *C. reticulata* (SM, 99.1%), *C. paradisi* (98.4%), ((*C. unshiu* X *C. sinensis*) X *C. reticulata*) X *C. reticulata* (98.1%), (*C. unshiu* X *C. sinensis*) X *C. reticulata* (98.0%), and (*C. unshiu* X *C. sinensis*) X *C. unshiu* (97.8%) showed superior activity to the other oils.

The NC group showed increases of more than 5.2- and 3.9-fold in the expression levels of *TRP-1* and *-2*, respectively, compared to the VE group (Figure 5c,d). Compared to the NC group, the kojic acid and essential oils revealed considerable inhibitory activities against the *TRP-1* and *-2* expression. More specifically, the essential oils of (*C. unshiu* X *C. sinensis*) X *C. reticulata* (56.4%), *C.* X *aurantium* (KA, 44.8%), and *C. maxima* (DA, 43.4%) exhibited more effective activity for *TRP-1* expression than kojic acid. *C.* X *aurantium* (NA, 68.7%), (*C. unshiu* X *C. sinensis*) X *C. unshiu* (68.1%), and (*C. unshiu* X *C. sinensis*) X *C. reticulata* (66.0%) showed a greater inhibitory effect of the *TRP-2* expression than the other essential oils.

The NC group showed a significant increase of approximately 3.0-fold in the *MC1R* expression compared to the VE group (Figure 5e). The essential oils of *C. unshiu* X *C. sinensis*, *C.* X *aurantium* (NA), *C. sinensis*, and *C.* X *latifolia* exhibited similar activity to kojic acid (about 61.1% reduction). In particular, *C. unshiu* X *C. sinensis* (about 64.3% reduction) exhibited superior anti-melanogenesis activity compared to PC.

This study confirmed that *C. junos*, *C. limon*, and *C.* X *latifolia* efficiently inhibited the tyrosinase activity and melanin overproduction. The expression levels of *Tyrosinase*, *TRP-1*, *TRP-2*, *MITF*, and *MC1R* were significantly suppressed by (*C. unshiu* X *C. sinensis*) X *C. reticulata* and *C. reticulata* (SM). The citrus cultivars bred based on *C. reticulata* [25,26] showed superior anti-melanogenesis activity in this study. The *C. reticulata*-based cultivars and other citrus cultivars were distinguished based on five chemical molecules, including limonene, farnesene, β-elemene, terpinen-4-ol, and sabinene, using GC–MS-based metabolomics.

### 2.3. Effects of Single Compounds

#### 2.3.1. Cell Viability

**Figure 6:**
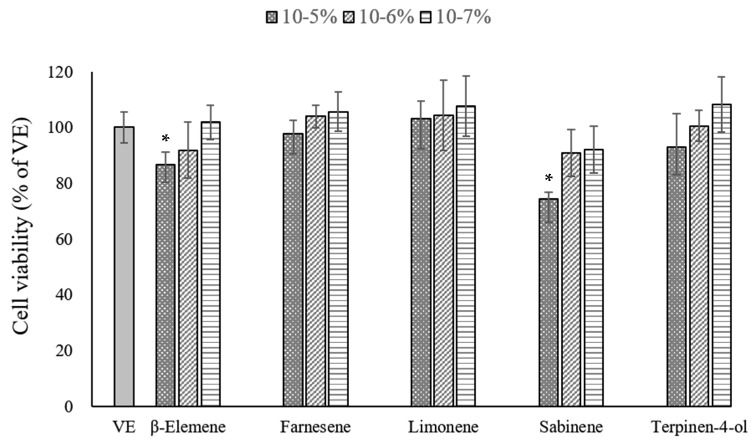
Cell viability assessed using the CCK assay. Values are presented as means ± standard deviations. Note: * *p* < 0.05 compared to VE. VE: vehicle group.

To identify the active compound for the anti-melanogenesis activity of *C*. *reticulata*-based cultivars, five compounds (limonene, farnesene, β-elemene, terpinen-4-ol, and sabinene) were assessed for their anti-melanogenesis activity. A CCK assay was performed on the five compounds to investigate their cytotoxicity for the B16BL6 cells, and the results are shown in Figure 6. The compounds exhibited toxicity in a dose-dependent manner. For B16BL6 melanoma cells, cell viability of greater than 80% was observed in the cells treated with each compound at 10^−7^–10^−6^%, indicating their non-cytotoxic effect. Therefore, further study of the anti-melanogenesis activity proceeded at a concentration of 10^−6^%.

#### 2.3.2. Effects of Single Compounds on Tyrosinase Activity

**Figure 7:**
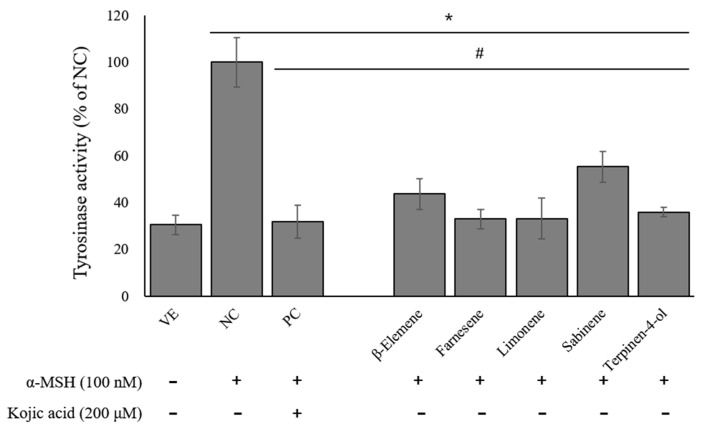
Effects of single compounds on tyrosinase activity. Values are presented as means ± standard deviations. Note: * *p* < 0.05 compared to VE; # *p* < 0.05 compared to NC. VE, vehicle group; NC, 100 nM α-MSH; PC, 200 µM kojic acid.

Figure 7 shows the effects of the selected five compounds on the tyrosinase release. The tyrosinase secretion was approximately 3.0-fold higher in the NC group than in the VE group. However, the treatment with kojic acid decreased the tyrosinase secretion by about 68.2% compared to the NC group. Similarly, each of these five compounds significantly reduced the tyrosinase secretion, with farnesene and terpinen-4-ol being the most potent by reducing the tyrosinase secretion by 67.2% and 64.2%, respectively.

#### 2.3.3. Melanin Content

**Figure 8:**
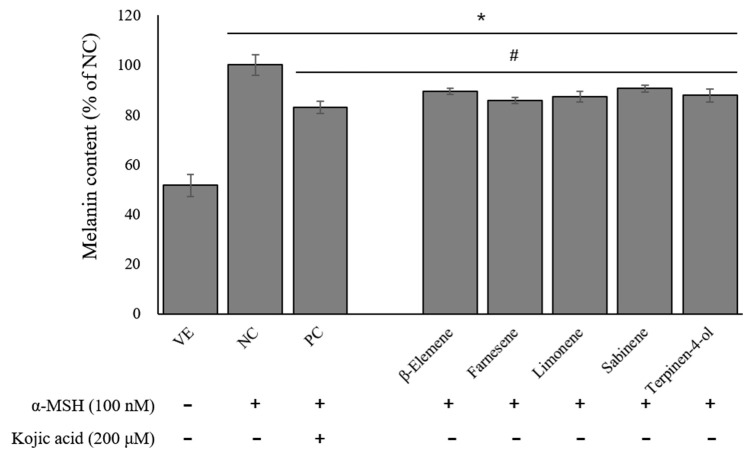
Effects of single compounds on melanin contents. Values are presented as means ± standard deviations. Note: * *p* < 0.05 compared to VE; # *p* < 0.05 compared to NC. VE, vehicle group; NC, 100 nM α-MSH; PC, 200 µM kojic acid.

The effects of the five compounds on the melanin content are presented in Figure 8. The NC group showed a significant increase in melanin production of nearly 2-fold compared to the VE group. The kojic acid inhibited the melanin production by about 16.9%, and the single compounds inhibited melanin production by about 9.4–14.3%. Among them, the inhibitory effect of farnesene was higher than others. This result indicates that farnesene is a potentially suitable ingredient for skin-whitening agents.

#### 2.3.4. Gene Expression

**Figure 9:**
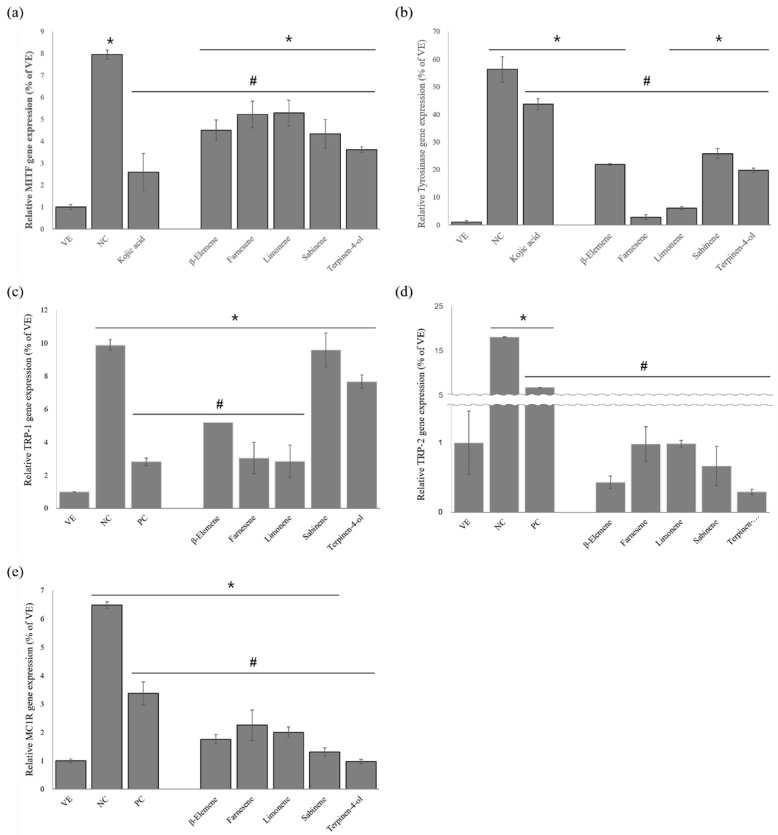
Effects of single compounds on *α*-MSH-induced B16BL6 cells: (**a**) *MITF* expression; (**b**) *Tyrosinase* expression; (**c**) *TRP-1* expression; (**d**) *TRP-2* expression; (**e**) *MC1R* expression. Values are presented as means ± standard deviations. Note: * *p* < 0.05 compared to VE; # *p* < 0.05 compared to NC. VE, vehicle group; NC, 100 nM α-MSH; PC, 200 µM kojic acid.

Figure 9 shows the anti-melanogenesis activities of the five single compounds. As shown in Figure 9a, the NC group showed an 8.0-fold increase in the *MITF* expression level compared to the VE group. The treatment with kojic acid inhibited the *MITF* expression level by about 67.4% compared to the NC group. The single compounds inhibited the *MITF* expression level compared to the NC group as follows: 54.5% for terpinen-4-ol, 45.5% for sabinene, 43.3% for β-elemene, 34.2% for farnesene, and 33.5% for limonene. Thus, the results show that terpinen-4-ol was the most effective among the single compounds.

As shown in Figure 9b, the NC group showed a remarkable increase in *Tyrosinase* expression compared to the VE group. The kojic acid suppressed this increase by 22.4%. The single compounds also inhibited the *Tyrosinase* secretion, with farnesene being the most effective at a 95.0% reduction rate.

As shown in Figure 9c, similar results were observed regarding the *TRP-1* expression. The five compounds exhibited anti-melanogenesis activity compared to the NC group. The NC group showed a 9.9-fold increase in *TRP-1* expression compared to the VE group, but this was significantly inhibited by the treatment with kojic acid and the single compounds. The kojic acid inhibited the *TRP-1* expression by 71.5% compared to the NC group, and limonene showed superior inhibitory activity among the single compounds, with reductions equaling 71.3%.

As shown in Figure 9d, the NC group showed a significant increase of 18.1-fold in the *TRP-2* expression level compared to the VE group. The treatment with kojic acid suppressed the *TRP-2* expression by 62.8% compared to the NC group. The single compounds also significantly suppressed *TRP-2* expression compared to the NC group, among which terpinen-4-ol was the most effective.

As shown in Figure 9e, the NC group showed a 6.5-fold increase in the *MC1R* expression compared to the VE group. The kojic acid and the single compounds significantly decreased the *MC1R* expression compared to the NC group. The kojic acid showed an inhibitory activity of 47.8%. Superior inhibitory activity was observed for terpinene-4-ol (84.8%).

## 3. Discussion

Several scientific studies on the anti-melanogenic properties of citrus oils have been published. Although the essential oils from plants such as bergamot, grapefruit, lemon, mandarin, and petitgrain are known for their cosmetic efficacy [27], the research is limited to certain cultivars. There are more than 1100 known cultivars of citrus fruits worldwide [28]. In addition, citrus crossbreeding is increasingly common to improve the fruit quality and develop new cultivars with novel traits [29]. Compared to the number of these citrus cultivars, the research on anti-melanogenesis is lacking. Therefore, this study evaluated anti-melanogenesis activities in 21 citrus cultivars, including the mandarin, orange, lemon, pummelo, and citron.

In this study, essential oils extracted from 21 citrus cultivars were evaluated for their anti-melanogenesis effects on B16BL6 melanoma cells. Overall, the essential oils of *C. reticulata*-based cultivars showed superior anti-melanogenesis activity among the 21 essential oils. The *C. reticulata*-based cultivars with superior effect include *C. junos*, *C. reticulata*, (*C. unshiu* X *C. sinensis*) X *C. reticulata*, and (*C. unshiu* X *C. sinensis*) X *C. reticulata* X *C. reticulata*. In addition, the essential oils of the *C. reticulata*-based cultivars showed a higher inhibitory effect on melanin production than kojic acid. Kojic acid (5-hydroxy-2-(hydroxymethyl)-4H-pyran-4-one) is well-known as a skin-whitening agent that is effective in regulating melanogenesis by inhibiting the catecholase activity of tyrosinase [30,31]. Because essential oils are fat-soluble, they have greater cell membrane permeability than the water-soluble kojic acid [32], which may explain their greater anti-melanogenesis activity. Therefore, *C. reticulata*-based cultivars can be considered potential whitening agents to prevent and treat hyperpigmentation diseases.

Melanin synthesis is mediated by melanocyte-specific enzymes such as *Tyrosinase*, *TRP-1*, and *TRP-2*, which are regulated by *MITF* [33]. The essential oils of (*C. unshiu* X *C. sinensis*) X *C. reticulata*, *C. reticulata*, *C. unshiu* X *C. sinensis*, and (*C. unshiu* X *C. sinensis*) X *C. reticulata* X *C. reticulara* decreased the expression levels of *MITF*, *Tyrosinase*, *TRP-1*, and *MC1R*. However, the results for the *TRP-2* expression suggest that the essential oils of *C.* X *aurantium* efficiently inhibited melanogenesis. Tyrosinase regulates the rate of oxidation of L-DOPA during melanin formation [34], indicating that melanogenesis mainly depends on the *Tyrosinase* expression; hence, *C. reticulata*-based cultivars should be more suitable for treating hyperpigmentation.

Citrus essential oils have been reported to be non-toxic; however, studies have shown that some citrus oils exhibit phototoxicity [35]. For example, bergamot, lime, lemon, and bitter orange oils are known to be phototoxic. The bergapten and furocoumarins in these essential oils have been reported to cause skin irritation when exposed to UV light [36]. Therefore, these essential oils and non-volatile compounds are restricted for use in some products [37]. The above compounds were not detected in the essential oils used in this study. Citrus essential oils have Generally Recognized As Safe (GRAS) status as assessed by the Food and Drug Administration (FDA) [38]. However, since no human subject studies exist, caution should be demonstrated when considering the concentration and recommended route of administration for use.

Essential oils and their isolated compounds are widely used in cosmetic ingredients as they impart benefits, such as pleasant fragrances and various other effects. In particular, cosmetic preparations are not required to contain an additional chemical preservative when they contain an essential oil or a single compound as an active agent because of the anti-bacterial and anti-fungal nature of essential oils [39]. However, essential oils should be used carefully because they can cause allergic reactions. The international organization the International Fragrance Association (IFRA) defined compounds with potential allergy-causing risks and suggested the appropriate concentrations for safe cosmetic products according to 12 categories [40]. Annex Ⅱ and Annex Ⅲ of the EU Cosmetics Regulations have defined twenty-six allergenic fragrances, and eighteen are found in essential oils [41]. Among them, citral, D-limonene, and linalool are detected in citrus essential oils (Figure 1). Limonene is easily oxidized when exposed to air and can cause skin irritation and sensitization. While limonene and linalool are specification compounds, citral is specified as a restricted compound. Therefore, citrus oils and citral should be applied in limited concentrations, depending on the cosmetic product categories. However, considering the number of application fields for individual ingredients of essential oils, they are generally safe to use in cosmetic products, despite their allergic reactions.

He et al. [42] reported that the essential oil of *Citrus maxima* cv. Guan Xi more effectively inhibited tyrosinase activity levels and melanin contents than kojic acid. Their study attributed this anti-melanogenesis activity to limonene, β-myrcene, β-pinene, ocimene, and β-copaene. The *C. maxima* oil used by He et al. accounted for 95.9% of the corresponding components, and the *C. maxima* oil used in this study had a higher portion of the corresponding components at 97.3%. Although the active compounds accounted for a lower portion, the anti-melanogenesis activity of (*C. unshiu* X *C. sinensis*) X *C. reticulata* oil, *C. reticulata* oil, and (*C. unshiu* X *C. sinensis*) X *C. reticulata* X *C. reticulata* oil used in this study was superior to the *C. maxima* oil. Thus, other constituents are believed to be contributing to the observed anti-melanogenesis activity.

*C. reticulata*-based cultivars and other citrus cultivars were distinguished based on five chemical molecules, including limonene, farnesene, β-elemene, terpinen-4-ol, and sabinene. Overall, all five compounds had anti-melanogenesis activity and significantly suppressed the *MITF*, *Tyrosinase*, *TRP-1*, and *MC1R* gene expression related to melanogenesis. However, only β-elemene, farnesene, and limonene significantly decreased the *TRP-2* gene. *TRP-1* and *TRP-2* are the major targets of *MITF*-induced melanogenic enzymes, but they undergo different mechanisms. *TRP-1* oxidizes dopachrome to 5,6-dihydroxyindole-2-carboxylic acid (DHICA) and carboxylates indole–quinone, which is converted to melanin, while *TRP-2* catalyzes the rearrangement of dopachrome into DHICA [43]. Therefore, among the five compounds, β-elemene, farnesene, and limonene are concluded to be more effective as dopachrome tautomerases in the melanin production pathway.

The active compound that contributed to the superior anti-melanogenesis activities of (*C. unshiu* X *C. sinensis*) X *C. reticulata*, *C. reticulata*, (*C. unshiu* X *C. sinensis*) X *C. reticulata* X *C. reticulara* was found to be terpinene-4-ol because of the presence of a hydroxyl group in the component [44]. Hsiao et al. [20] evaluated the anti-melanogenesis activity of 11 single compounds including terpinen-4-ol. Among them, the melanogenesis inhibitory activity of thymol was the most superior. This is due to the coexistence of hydroxyl and methoxy groups, which heavily influences the melanogenesis inhibitory activities [45].

Notably, among the five compounds except limonene, all are trace components in the essential oils. Several studies have reported that minor components have excellent physiological activities [46,47]. In addition, the essential oils of the citrus cultivars showed superior activity to the single compounds. These notable differences in efficacy suggest that the minor constituents in essential oils contribute a synergistic effect that improves the overall benefits of the essential oil [48].

The anti-melanogenesis activity regulates melanin production by inhibiting the expression and activity of tyrosinase and suppressing the distribution of melanosomes [49]. This study, however, showed that the tyrosinase activity is not always associated with tyrosinase mRNA expression (Figure 2 and 5b). As such, compounds that could regulate melanin synthesis by affecting tyrosinase activity without changes in mRNA expression are known to regulate the activity of melanogenic enzymes at post-translational levels [50]. Ando et al. [51] found that the melanosomes that synthesize melanin pigments change the tyrosinase mRNA level but not the tyrosinase activity.

## 4. Materials and Methods

### 4.1. Plant Materials

Peels from 21 citrus cultivars were collected in December 2019 from Jeju Island. Four cultivars were collected from a tangerine orchard (33°17’27.70’’ N, 126°41’38.7’’ E), and 17 cultivars were collected from the National Institute of Horticultural and Herbal Science (33°18’05.60’’ N, 126°36’43.60’’ E). Voucher specimens were deposited in the National Institute of Forest Science herbarium. Details on the samples are presented in Table 2.

### 4.2. Extraction of Essential Oils

The essential oils from the citrus cultivar peels were extracted by hydro-distillation using a Clevenger-type apparatus. First, 6.0 L of distilled water was poured into a 10-L round-bottom flask containing 1.0 kg of citrus peel, and the flask was then placed on a digital heating mantle (MS-DM 608, MTOPS^®^, Misung Scientific Co., Ltd., Yangju, Korea). The essential oils were extracted from the peels at 10 ± 2 °C until no more essential oil could be obtained. The collected essential oil was dried over anhydrous sodium sulfate (Samchun Chemical Co., Ltd., Seoul, Republic of Korea) and was filtered through a 0.45-µm membrane disk filter. The obtained essential oils were stored in sealed dark vials at 4 °C until use.

### 4.3. Gas Chromatography–Mass Spectrometry

A Trace 1310/ISQ-LT gas chromatography–mass spectrometry system (GC–MS, Thermo Fisher Scientific, Waltham, MA, USA) equipped with a VF-5MS capillary column (60 m × 0.25 mm × 0.25 µm; Thermo Fisher Scientific Inc., Waltham, MA, USA) and a flame ionization detector were used to determine the components of the essential oils. Helium was used as the carrier gas at a constant flow rate of 1.0 mL/min. The injector temperature was set at 250 °C with a split ratio of 1:20. The oven temperature was programmed as follows: keep at 50 °C for 5 min, increase to 65 °C at 10 °C/min, increase to 210 °C at 5 °C/min, increase to 325 °C at 20 °C/min, and then keep at 325 °C for 10 min. For flame ionization detection, the temperature was 300 °C, the airflow was 350.0 mL/min, the hydrogen flow was 35.0 mL/min, and the make-up gas flow was 40.0 mL/min. The mass spectrometer was operated in electron ionization mode at 70 eV; the mass interface temperature was 250 °C, and the ion source temperature was 270 °C. The mass spectra were recorded in the range of 35–550 amu.

The essential oil components were identified by comparing their retention times and mass spectra obtained from the GC–MS analysis with those obtained from the NIST libraries (National Institute of Standards and Technology, USA). The Kovats index was used to identify the individual components by comparing their relative retention times with those of an *n*-alkane mixture (C_8_–C_30,_ Sigma-Aldrich, St. Louis, MO, USA).

### 4.4. Cell Culture

The mouse melanoma B16BL6 cells were obtained from the Korea Cell Line Bank (Seoul, Korea). The B16BL6 cells (KCLB No. 80006) were cultured with 10% fetal bovine serum (Gibco, Carlsbad, CA, USA), 1% penicillin–streptomycin (Gibco), and 0.4 µL/mL Plasmocin™ (pH 7.4; Invivogen, San Diego, CA, USA), and were supplied with Dulbecco’s modified Eagle’s medium (Welgene Inc., Gyeongsan, Republic of Korea) at 37 °C in a 5% CO_2_ incubator (Panasonic, Kadoma, Osaka, Japan).

### 4.5. Cytotoxicity

To evaluate the cytotoxicity of the essential oils or single compounds to B16BL6 cells, a cell counting assay (CCK-8; DoGenBio Co., Ltd., Seoul, Republic of Korea) was performed according to a previously reported protocol [52]. The B16BL6 cells were seeded at a density of 1 × 10^4^ cells/well in 96-well plates and incubated at 37 °C for 24 h. The cells were treated with 10^−7^%–10^−5^% of essential oils or single compounds in a medium at 37 °C for 24 h. After treatment, the cells were washed with Dulbecco’s phosphate-buffered saline (Gibco), and CCK-8 solution was added to each well. The plates were incubated at 37 °C for 1 h. The absorbance of the solution was measured at 450 nm using a microplate reader (Epoch, Bio Tek Instruments Inc., Winooski, VT, USA).

### 4.6. Tyrosinase Activity Assay

The B16BL6 melanoma cells were seeded at 1 × 10^5^ cells/well in a 6-well plate and were pre-cultured at 37 °C in a 5% CO_2_ incubator. The B16BL6 cells were stimulated with α-MSH (100 nM) for 1 h, which was followed by treatment with kojic acid (TCI Co., Ltd., Tokyo, Japan), essential oils, or single compounds for 48 h. The cells were lysed in a lysis buffer (0.1 M sodium phosphate buffer (pH 6.8; Welgene), 1% Triton X-100 (Sigma-Aldrich), 0.2 mM phenylmethylsulfonyl fluoride (PMSF; Thermo Fisher Scientific)) and were then centrifuged at 10,000 rpm for 15 min. The cells were vortexed in a protein extraction solution, and then the lysate was centrifuged at 10,000 rpm for 15 min. After centrifugation, 60 µL of the supernatant was transferred into the 96-well plate, and 140 µL of 2 mg/mL 3,4-dihydroxy-L-phenylalanine (L-DOPA; Sigma-Aldrich) was added. The plates were then incubated at 37 °C for 1 h, and the absorbance was measured at 490 nm. The supernatant was aspirated, and the cell pellet was set aside to determine the melanin content.

### 4.7. Determination of Melanin Content

The B16BL6 melanoma cells were cultured at 1 × 10^5^ cells/well in a 6-well plate and were then treated with 100 nM α-MSH, kojic acid, essential oils, or single compounds for 48 h. After centrifugation, the cell pellets were dissolved in 1 N NaOH (Welgene) for 1 h at 80 °C. The mixture was then vigorously vortexed to solubilize the melanin pigment. The melanin content was measured by absorbance at 405 nm on a microplate reader

### 4.8. Quantitative Real-Time Polymerase Chain Reaction

The B16BL6 cells were grown on six-well plates (1 × 10^5^ cells/well) for 24 h. The cells were stimulated with 100 nM α-MSH for 1 h and were then treated with 200 µM kojic acid, 21 essential oils, or the single compounds for 48 h. The total RNA was isolated using the TRIzol reagent (Invitrogen, Waltham, MA, USA) and quantified using a Nanodrop 2000 spectrophotometer (Thermo Fisher Scientific). The total RNA was reverse-transcribed into first-strand complementary DNA (cDNA) using Moloney murine leukemia virus reverse transcriptase (M-MLV; Invitrogen) and random primers (9-mer; Takara Bio Inc., Shiga, Japan). Each cDNA sample was amplified with 2 × SYBR^®^ Premix Ex Taq™ (Takara Bio Inc.) and 3 pmol of each primer. The oligonucleotide primers used for PCR amplification that were obtained from the target cellular RNA are listed in Table 3. The real-time PCR assay was performed by using a CFX96 real-time PCR system (Bio-Rad Laboratories, Inc., Hercules, CA, USA). The PCR amplification process comprised 40 cycles of denaturation at 95 °C for 10 s, annealing at 55 °C for 30 s, and extension at 95 °C for 5 s. The C_t_ gene expression values were normalized to the corresponding values for the *β*-actin gene expression using Bio-Rad CFX Maestro.

### 4.9. Statistical Analysis

Each experiment was performed at least three times, and the results were expressed as the means ± standard deviations. A repeated one-way analysis of variance was performed using the Statistical Package for the Social Sciences (ver. 24.0; IBM, Republic of Korea) program, and *p* values lower than 0.05 were considered statistically significant.

## 5. Conclusions

More than 1100 citrus cultivars have been cultivated through hybridization. However, only some cultivars are used therapeutically due to the lack of anti-melanogenesis activity studies on citrus cultivars. According to a previous study, three citrus fruits, mandarin (*C. reticulata*), citron (*C. medica*), and pummelo (*C. maxima*), are considered original species [53]. This study was conducted with 21 citrus essential oils, including the three original species, to utilize citrus oils widely. The components of the essential oils were similar in all 21 cultivars, but each cultivar exhibited different melanogenesis inhibitory activity levels. Among the 21 cultivars, *C. junos*, *C. reticulata*, (*C. unshiu* X *C. sinensis*) X *C. reticulata*, and (*C. unshiu* X *C. sinensis*) X *C. reticulata* X *C. reticulata* showed superior inhibitory effects on melanogenesis production. It was found that the cultivars with superior efficacy were *C. reticulata*-based cultivars, and the active components in these essential oils were identified to be β-elemene, farnesene, and limonene. These results suggest that *C. reticulata*-based cultivars, *β*-elemene, farnesene, and limonene are potentially suitable for use in cosmetics and medicines against skin hyperpigmentation. Although it was confirmed that there was no cytotoxicity in this study, further toxicity evaluations are needed before their use as tyrosinase inhibitors. In addition, more studies are needed on the inhibitory mechanisms of active anti-melanogenesis compounds to prove the association between the biologically active compounds of *C. reticulata* and anti-melanogenesis.

## Figures and Tables

**Table 1:** Chemical compositions of essential oil from citrus cultivar peels.

KI ^a^	Compound Name	Area %																				
		BY	DA	FC	JI	KA	KU	KY	LI	MW	NA	PL	PO	PU	SE	SH	SM	RU	ST	TS	YN	YU
926	α-Pinene	0.30	0.26	2.00	0.45	0.41	0.32	0.32	1.05	0.61	0.46	1.64	0.68	0.19	0.55	0.42	0.67	0.38	0.49	0.61	0.34	1.06
965	Sabinene	0.05	–	0.07	0.11	0.03	–	0.03	0.13	0.01	–	0.21	0.08	–	–	0.16	0.02	0.29	0.63	0.20	0.04	0.01
970	β-Pinene	0.85	–	1.62	1.98	0.86	–	–	6.03	0.25	0.21	7.55	0.26	0.22	0.12	0.04	0.34	0.14	0.36	0.27	–	0.50
986	β-Myrcene	19.48	21.62	–	1.17	1.23	1.34	1.23	0.95	1.21	1.09	0.84	1.17	28.09	1.31	1.36	1.27	1.25	1.05	1.18	1.31	1.25
1034	m-Cymene	–	–	0.83	–	–	–	–	1.33	0.27	0.21	1.90	0.13	0.04	0.10	–	0.39	0.04	0.05	1.29	0.01	0.63
1041	D-Limonene	77.13	76.33	59.19	92.35	95.19	97.19	96.49	69.01	90.59	90.40	50.88	90.50	68.79	91.96	94.24	89.32	93.47	91.11	89.21	95.74	77.98
1047	cis-β-Ocimene	0.13	0.06	1.16	0.10	0.01	–	–	0.04	–	–	0.04	–	–	–	0.10	–	–	–	–	–	–
1058	trans-β-Ocimene	0.52	0.40	1.76	0.38	0.10	0.04	0.07	0.10	0.07	0.19	0.10	0.06	0.20	0.32	0.44	0.10	0.44	0.14	0.20	0.04	0.31
1068	γ-Terpinene	0.07	0.02	27.29	0.11	0.06	0.01	0.12	9.73	4.57	5.03	17.61	5.05	0.08	3.42	0.37	5.49	0.65	0.84	3.89	0.17	11.53
1090	Terpinolene	0.06	0.14	1.25	0.11	0.19	0.04	0.06	1.01	0.26	0.37	1.92	0.26	0.21	0.24	0.12	0.33	0.31	0.23	0.27	0.09	0.70
1099	Linalool	0.42	0.10	0.06	0.73	0.57	0.14	0.11	0.37	0.42	0.17	0.72	0.71	0.18	0.33	0.45	0.12	0.23	0.95	0.29	0.35	1.97
1146	β-Terpineol	0.02	0.03	0.01	0.03	0.09	0.05	0.08	0.11	0.05	0.12	0.13	0.02	0.06	0.17	0.10	0.03	0.07	0.06	0.04	0.12	0.10
1179	Terpinen-4-ol	0.13	0.04	0.46	0.24	0.13	0.03	0.21	1.19	0.11	0.15	1.69	0.17	0.08	0.13	0.71	0.11	1.14	1.78	0.66	0.31	0.34
1194	α-Terpineol	0.28	0.19	0.66	0.49	0.58	0.28	0.45	3.28	0.37	0.77	5.85	0.26	0.37	0.96	0.51	0.26	0.57	0.51	0.41	0.58	1.01
1243	(Z)-Citral	–	–	0.26	–	–	–	–	1.44	–	–	1.70	–	0.08	–	–	–	–	–	–	–	–
1273	(E)-Citral	–	–	0.26	–	–	–	–	2.17	–	–	2.43	–	0.07	–	–	–	–	–	–	–	–
1395	β-Elemene	0.03	0.01	-	0.97	0.05	0.03	0.02	-	0.49	0.09	0.10	0.01	-	0.01	-	0.72	0.02	0.06	0.14	0.01	0.05
1457	β-Farnesene	0.03	–	–	–	–	–	–	–	–	–	–	–	–	–	–	–	0.01	0.01	0.05	–	0.21
Monoterpene hydrocarbons		97.29	89.97	97.84	97.93	98.15	98.38	83.69	95.05	98.63	96.82	98.13	98.01	98.85	98.12	98.38	98.44	96.37	97.41	97.47	98.16	98.95
Oxygenated monoterpenes		2.36	9.10	1.18	1.62	1.45	1.10	13.43	3.48	0.85	1.51	1.60	1.04	0.61	1.57	1.10	1.33	1.71	2.01	2.18	0.54	0.52
Sesquiterpene hydrocarbons		0.16	0.29	0.02	0.37	0.21	0.09	1.46	0.96	0.22	1.35	0.10	0.81	0.18	0.11	0.09	0.04	0.53	0.25	0.13	1.11	0.23
Oxygenated sesquiterpenes		0.02	0.49	–	–	0.06	0.06	1.17	0.16	–	0.08	0.04	0.03	0.01	–	0.06	–	0.03	0.10	–	0.02	0.08
Unknown compounds		0.15	0.14	1.00	0.11	0.08	0.35	0.22	0.37	0.30	0.25	0.13	0.15	0.37	0.21	0.35	0.17	0.26	0.21	0.23	0.20	0.21
Total		100	100	99	100	100	100	100	100	100	100	100	100	100	100	100	100	99	100	100	100	100

^a^ Kovats retention index was experimentally determined using a VF-5MS column with a homologous series of C_8_–C_30_ alkanes. BY, *C. platymamma*; DA, *C. maxima*; FC, *C. medica*; JI, *C. sunki*; KA, *C.* X *aurantium*; KU, *C. japonica*; KY, *C. unshiu* X *C. sinensis*; LI, *C. limon*; MW, *C. reticulata*; NA, *C.* X *aurantium*; PL, *C.* X *latifolia*; PO, *C. reticulata*; PU, *C. maxima*; RU, *C. paradisi*; SE, *C.* X *aurantium*; SH, (*C. unshiu* X *C. sinensis*) X *C. reticulata*; SM, *C. reticulata*; ST, ((*C. unshiu* X *C. sinensis*) X *C. reticulata*) X *C. reticulata*; TS, (*C. unshiu* X *C. sinensis*) X *C. unshiu*; YN, *C. sinensis*; YU, *C. junos*.

**Table 2:** Summary of cultivar information for citrus peels.

No.	Sample Name	Abbreviation	Voucher Specimen
1	*Citrus japonica* Thunb.	KU	WTFRC10032742
2	*Citrus junos* Siebold ex Tanaka	YU	WTFRC10032743
3	*Citrus limon* (L.) Osbeck ‘Lisbon’	LI	WTFRC10033803
4	*Citrus maxima* (Burm.) Merr.	DA	WTFRC10032725
5	*Citrus maxima* (Burm.) Merr. ^a^	PU	WTFRC10032744
6	*Citrus medica* L. ^b^	FC	WTFRC10033804
7	*Citrus paradisi* Macfad. ‘Redblush’	RU	WTFRC10032741
8	*Citrus platymamma* hort. ex Tanaka	BY	WTFRC10032726
9	*Citrus reticulata* Blanco ^c^	MW	WTFRC10032727
10	*Citrus reticulata* Blanco ‘Ponkan’	PO	WTFRC10032734
11	*Citrus reticulata* Blanco ^d^	SM	WTFRC10032740
12	*Citrus sinensis* (L.) Osbeck ‘Navel’	YN	WTFRC10032732
13	*Citrus sunki* (Hayata) Yu. Tanaka	JI	WTFRC10032733
14	*Citrus* X *aurantium* L. ^e^	NA	WTFRC10032737
15	*Citrus* X *aurantium* L. ^f^	KA	WTFRC10032735
16	*Citrus* X *aurantium* L. ^g^	SE	WTFRC10032729
17	*Citrus* X *latifolia* (Yu. Tanaka) Yu. Tanaka	PL	WTFRC10032736
18	*Citrus unshiu* X *Citrus sinensis*	KY	WTFRC10032739
19	(*Citrus unshiu* X *Citrus sinensis*) X *Citrus reticulata*	SH	WTFRC10032728
20	(*Citrus unshiu* X *Citrus sinensis*) X *Citrus unshiu*	TS	WTFRC10032731
21	(*Citrus unshiu* X *Citrus sinensis*) X *Citrus reticulata* X *Citrus reticulata*	ST	WTFRC10032730

^a^ Synonym of *C. grandis* (L.) Osbeck. ^b^ Synonym of *C. medica* var. sarcodactylus (Siebold ex Hoola van Nooten) Swingle. ^c^ Synonym of *C. unshiu* (Yu.Tanaka ex Swingle) Marcow, “Miyagawa-wase”. ^d^ Synonym of *C. unshiu* (Yu.Tanaka ex Swingle) Marcow. ^e^ Synonym of *C.* X *natsudaidai* (Yu.Tanaka) Hayata. ^f^ Synonym of *C.* X *benikoji* Yu.Tanaka. ^g^ Synonym of *C.* X *tangelo* J.W.Ingram and H.E.Moore.

**Table 3:** Oligonucleotide primer sequences used for a quantitative real-time polymerase chain reaction.

Gene	Primer Sequence (5′–3′)	NCBI No.
*TRP-1*	F: 5′-GCT GCA GGA GCC TTC TTT CTC-3′ R: 5′-AAG ACG CTG CAC TGC TGG TCT-3′	AL670884
*TRP-2*	F: 5′-GGA TGA CCG TGA GCA ATG GCC-3′ R: 5′-CGG TTG TGA CCA ATG GGT GCC-3′	X63349
*Tyrosinase*	F: 5′-GGC CAG CTT TCA GGC AGA GGT-3′ R: 5′-TGG TGC TTC ATG GGC AAA ATC-3′	D00131
*MITF*	F: 5′-AGC GTG TAT TTT CCC CAC AG-3′ R: 5′-TAG CTC CTT AAT GCG GTC GT-3′	BC108977
*MC1R*	F: 5′-TGA CCT GAT GGT AAG TGT CAG C-3′ R: 5′-ATG AGC ACG TCA ATG AGG TT-3′	NM_008559
*β-actin*	F: CAG GTC ATC ACT ATT GGC AA R: AGG TCT TTA CGG ATG TCA AC	AY618569

## Data Availability

Not applicable.
